# Liver X receptor-β improves autism symptoms via downregulation of β-amyloid expression in cortical neurons

**DOI:** 10.1186/s13052-016-0249-4

**Published:** 2016-05-06

**Authors:** Ji-Xiang Zhang, Jun Zhang, Ye Li

**Affiliations:** Department of Clinical Psychology, Linyi People’s Hospital, Linyi, 276000 China; Department of Children’s Rehabilitation, Linyi People’s Hospital, No.27 East Jiefang Road, Linyi, 276000 China; Department of Outpatient Operation Room, Linyi People’s Hospital, Linyi, 276000 China

**Keywords:** Liver X receptor, T0901317, β- amyloid protein, Autism, Behavioral observation

## Abstract

**Background:**

We study the effect of liver X receptor β (LXRβ) on β-amyloid (Aβ) peptide generation and autism behaviors by conducting an animal experiment.

**Methods:**

In autistic mice treated with LXRβ agonist T0901317, enzyme linked immunosorbent assay was used to measure Aβ in brain tissue homogenates. Western blot was used to detect Aβ precursors, Aβ degradation and secretase enzymes, and expression of autophagy-related proteins and Ras/Raf/Erkl/2 signaling pathway proteins in brain tissue. Changes in autism spectrum disorder syndromes of the BTBR mice were compared before and after T0901317 treatment.

**Results:**

Compared with the control group, autistic mice treated with LXRβ agonist T0901317 showed significantly lower Aβ level in brain tissue (*P* < 0.05), significantly higher Aβ degradation enzyme (NEP, IDE proteins) levels (all *P* < 0.05), significantly lower Aβ secretase enzyme BACE1 protein level (*P* < 0.05), and significantly lower Ras, P-C-Raf, C-Raf, P-Mekl/2, P-Erkl/2 protein levels (all *P* < 0.05). BTBR mice treated with T0901317 showed improvements in repetitive stereotyped behavior, inactivity, wall-facing standing time, self-combing time and center stay time, stayed longer in platform quadrant, and crossed the platform more frequently (all *P* < 0.05).

**Conclusions:**

LXRβ could potentially reduce brain Aβ generation by inhibiting Aβ production and promoting Aβ degradation, thereby increasing the expression of autophagy-related proteins, reducing Ras/Raf/Erkl/2 signaling pathway proteins, and improving autism behaviors.

## Background

Autism spectrum disorder (ASD), also known as autism, was discovered and named by Kanner in 1943 [1]. Autism is a pervasive developmental disorder in the neurological system [2]. Studies reported that the incidence rate is 0.4 % in infants in China, and in Korea, the prevalence of autism was estimated to be 2.64 % in school-age children [3, 4]. The clinical manifestations of autism include interpersonal barriers, rigid perceptions, verbal communication disorders, and stereotyped behaviors [5]. The incidence rate of autism continues to increase in modern society, bringing great mental and economic burden to the society and affected families [6]. However, the etiology for autism remains poorly understood, it was believed that both environmental and genetic factors were involved [4, 7]. Nevertheless, few studies have examined the development of autism from the molecular point of view.

Liver X receptors (LXRs) are ligand-activated transcription factors that belong to the nuclear hormone-receptor super family with two LXR isoforms: LXRα and LXRβ [8, 9]. LXRα and LXRβ were demonstrated to share approximately 78 % amino acid sequence identity in both DNA- and ligand-binding domains and appear to bind ligands with similar affinities [10]. Previous study focused on LXRα and LXRβ knockout mice also revealed that LXRα plays an crucial role in cholesterol homeostasis, whereas LXRβ has important functions in the immune system and central nervous system (CNS) [11]. Besides, the LXRβ serves important functions in the development of the CNS, and regulation of cholesterol absorption, discharge and transformation by regulating downstream genes [12, 13]. T0901317, used in our study, is a chemically synthesized ligand of LXR, which can activate LXR to cause increased expression of downstream gene ABCA1 and decreased β-amyloid (Aβ) level [14]. Aβ is a soluble polypeptide, a product of amyloid precursor protein (APP) degradation by β- and γ- secretase enzyme, which generally consists of 39–43 amino acids and exists in the form of Aβ _1–40_ and Aβ _1–42_ in human body [15, 16]. Under normal circumstances, Aβ is in soluble state and is nontoxic. However, under pathological conditions, Aβ aggregates to form oligomers, polymers, fibrillar structure, and even plaque. Researches generally believed that Aβ aggregation is the main cause of neuronal degeneration, apoptosis, and dementia [17]. Studies have shown that LXR activation will lower the level of the cholesterol and Aβ in neurons [8], but the mechanism for which LXRβ modifies autism behavior remains less studied. This article will examine the impact of LXRβ on Aβ generation and its effects and mechanism on autism behaviors.

## Methods

### Ethical statement

Animal experiments were conducted in strict accordance with the approved animal protocols and guidelines established by Medicine Ethics Review Committee for animal experiments of Linyi People’s Hospital.

### Experimental animals and grouping

A total of 32 adult BTBR mice aged 8–10 week and 16 B6 mice (C57BL/6) (BTBR mice were mice model with autistic behavior; B6 mice were normal healthy mice, purchased from Beijing Experimental Animal Center, China), which were 50 % male and weighted 20–25 g, were divided into: (1) the experimental group: 16 BTBR mice fed with agonist T0901317 (20 mg/kg/day, purchased form Cayman Company) which dissolved in dimethyl sulfoxide (DMSO); (2) the control group: 16 BTBR mice fed with DMSO of the same volume sans T0901317; and (3) the normal control group: 16 B6 mice fed with DMSO with the same volume sans T0901317. After seven consecutive days of gavage followed by fasting overnight, 8 mice in each group were randomly selected and executed the next day between 8:00 am to 4:00 pm. After intraperitoneal injection of 10 % water and chloral (0.4 g/kg, the lower left i.p.) anesthesia, the mice were then decapitated, and brains were harvested into liquid nitrogen and stored at −70 °C for measurement of Aβ. The remaining mice were placed into the conduct laboratory for one hour of acclimatization, and then used for autistic behavioral test.

### Enzyme linked immunosorbent assay (ELISA) measurement of Aβ protein in brain tissues

About 80 mg brain tissue was weighed and homogenates were prepared according to the methods by Howland DS et al. The Aβ radioimmunoassay kit (Bo Yan Biological Technology Co., Ltd. Shanghai) was used to determine the content of Aβ in the brain tissue samples. A 100 μL of homogenate was diluted by 5-fold with 1 mol/L Tris–HCl (PH 8.0) and mixed well, from which a 100 μL sample was taken for measurement. The standard product is diluted with PBS buffer to various concentrations and added antiserum and ^125^ I-Aβ protein, mixed well, stayed at 4 °C for 24 h and added separating agent at room temperature for 20 min, then centrifuged at 4 °C 3500 rpm for 20 min with the supernatant discarded. The cpm value was measured using γ counter and plotted for standard curves. The same protocol was then applied on the sample to calculate the positivity rates, which was used to determine the content of the Aβ in picograms per milliliter of sample volume (pg/ml) according to the standard curve.

### Western blot analysis

Brain tissue protein of LXRβ agonist T0901317 treated mice was extracted and tested for the changes in Aβ precursors APP, the Aβ-degrading enzymes (neutral endopeptidase, NEP, insulin-degrading enzyme, IDE), the β-secretase enzyme (BACE1) that cut the APP to generate Aβ, and autophagy-related protein (LC3-I, LC3-II, autophagy substrate protein P62, etc.), and to test the phosphorylated Ras/Raf/ERK1/2 protein expression. Western blots were performed according to the method described previously [18, 19]. The prepared protein sample was separated by SDS-PAGE electrophoresis (for BACE1, LC3-I, LC3-II, P62, we used 15 % gel; for APP, NEP, IDE, we used 8 % gel; for Ras, Raf, MEK1/2, ERK1/2 we used 10 % gel). The test was run with a sample concentration of 20–60 μg/L and under 80-120 V, the electrophoresis terminated at the first appearance of the bromophenol blue. The gel was removed, and placed in the order of filter, nitric acid cellulose membrane, gel, filter from bottom to top, and expelled air bubbles in between, and placed in electrophoretic transfer membrane device at 100 V for 1.5 h of transmembrane. Then, the nitrocellulose filter was removed and washed with PBST, added 5 % skim milk solution, shaken at room temperature and blocked for 2 h. The primary antibody was diluted with 5 % skim milk or 5 % BSA to the appropriate concentration (Anti-β-actin (Santa cruz, sc-47778, 1: 400); anti- IDE (Abeam, ab32216, l: 5000); anti-NEP (R & D, AF1126, 0.2 μg/ml); anti-APP (Sigma, A8717, 1: 20000); anti-BACEl (Abeam, ab2077, l: 1000); phospho-Mekl/2, phospho-C-Raf, Ras, phospho-ERKl/2 (Cell Signaling Technology, USA)). The membrane was washed with PBST for 10 min × 4 times and the secondary antibody was diluted in blocking buffer (1: 4000) and shaken at room temperature for 2 h, following, the membrane was washed with PBST, 10 min × 4 times, colored by chemiluminescence (ECL). Then the molecular weight of the target band and net optical density value of the sample was quantitatively analyzed using gel image processing system Image J (National Institutes of Health, Bethesda, MD, USA), and standardized with β-actin concentrations.

### Stereotyped behavior

Stereotyped behavior was assessed using blinded evaluation using 60 min video recording of mouse activity. Mice were placed into a round Plexiglas cylinder (12 inch tall and 8 inch in diameter) individually, and all the mice were admitted to explore freely. Mice ID, status, and body weight were recorded, and changes of behaviors including rotation, rears, sniffing, grooming and exploratory activity were observed. Two raters watched these recording and independently rated stereotyped behavior based on the animal stereotyped behavior score table and data summary table mentioned by Kelley AE [20].

### Open-field (OF) test

Experiment mice were transported to the laboratory around one hour in advance to adapt to the experimental environment. Experiment staff recorded ID, date and status of the mice, and cleaned experimental device with 75 % ethanol and water, and dried with paper towels, then released the mice (back facing the experimenter) from the cage into the center of the OF device, and used video recording system to record the activity of mice in the OF for a total of 15 min. The recorded indicators include total distance (total activity), average speed, rest time, activity time, the number of activities, regional distribution index (edges, corners, surrounding, center, grooming, grooming frequency and time).

### Water maze test

The experiment was divided into two parts. (1) Navigation test: experiment mice were allowed to free swimming for 2 min during the first day to adapt to the environment. Starting from the second day, the water maze was artificially divided into four quadrants. The platform was placed in a random quadrant and standing 1 cm above the water level and decorated with bright-color flags in order to attract the attention of mice. Mice were placed into the four quadrants facing the wall. Computer was used to monitor and record the route, distance and time the mice used to find and climb the platform from the fourth day. For mice that failed to find the platform in 2 min, they were drawn to the platform, and the incubation period was recorded as 2 min. (2) Space search experiment: the platform was removed in the fifth day, mice were placed into the water at a random point facing the wall, computer recorded the times of swimming in each quadrant and number of crossing the platform within 90s, and searched platform trajectory.

### Statistical analyses

We used SPSS 21.0 statistical software (SPSS Inc., Chicago, IL) for statistical analyses. Data were expressed as mean ± standard deviation ($\overline{\mathrm{x}}$ ± s). The one-way analysis of variance (ANOVA) was used to compare among multiple groups, *t*-test was used to compare between two groups. *P* < 0.05 was considered statistically significant.

## Results

### Comparison of brain Aβ

**Fig. 1:**
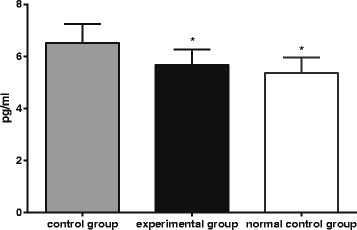
ELISA of Aβ in mice brain tissue treated with LXRβ agonist T0901317. Note: ELISA, enzyme linked immunosorbent assay; * compared with the control group, *P* < 0.05

Figure 1 and Table 1 show that, compared with the control group not treated with T0901317 (6.51 ± 0.73 pg/mg), the normal control group (5.37 ± 0.59 pg/mg) had significantly lower Aβ expressions (*P* = 0.004), suggesting Aβ is higher in the brain of autistic mice than normal B6 mice. After LXRβ agonist T0901317 processing, Aβ in brain tissue of mice in the experimental group (5.66 ± 0.60 pg/mg) was significantly lower compared with the control group not treated with T0901317 (6.51 ± 0.73 pg/mg).

**Table 1:** Aβ in mice brain tissue (pg/mg)

Packet	*N*	Aβ (pg/mg)
Control group	8	6.51 ± 0.73
Experiment group	8	5.66 ± 0.60^*^
Normal control group	8	5.37 ± 0.59^*^

^*^compared with the control group, *P* < 0.05

### Changes in brain Aβ secretion enzyme and Aβ degradation enzyme

**Fig. 2:**
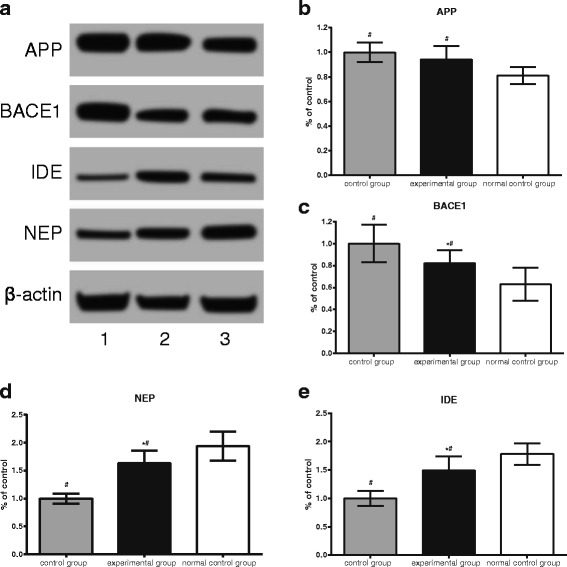
Western blot assays of changes in APP, BACE1, NEP, and IDE in the brains of three groups of mice. Note: APP: Beta Amyloid Precursor Protein; NEP: neprilysin; IDE: Insulin-degrading Enzyme; BACE1: beta-Secretase 1. Note: ^#^, indicates comparison with the normal control group, *P* < 0.05; *, indicates a comparison with the control group, *P* < 0.05; (**a**) Western blot assays of changes in APP, BACE1, NEP, and IDE in the brains of three groups of mice, 1 represented the control group, 2 represented the experimental group, 3 represented the normal control group; (**b**-**e**) changes in APP, BACE1, NEP, and IDE in the brains of three groups of mice

Figure 2 shows that, compared with the normal control group, the BTBR mice treated with the same volume of DMSO sans T0901317 in control group had significantly increased APP and BACE1 proteins, and significantly decreased Aβ degradation enzyme (NEP, IDE) protein levels (all *P* < 0.05). Compared with the control group, the autistic mice treated with T0901317 in experimental group had no significant difference in APP levels (*P >* 0.05), but increased Aβ degrading enzyme (NEP, IDE) levels (both *P* < 0.05), and lower BACE1 protein levels (*P* < 0.05).

### Autophagy-related protein

**Fig. 3:**
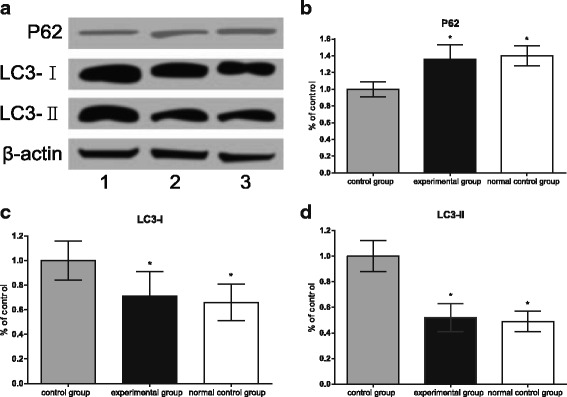
Western blot assays of changes in P62, LC3-I, LC3-II among three groups of mice brain; (**a**) Western blot assays of changes in P62, LC3-I, LC3-II in the brains of three groups of mice, 1 represented the control group, 2 represented the experimental group, 3 represented the normal control group; (**b**-**d**) changes in P62, LC3-I and LC3-II in the brains of three groups of mice. Note: P62: Protein 62; LC3-I: microtubule-associated Protein 1 light chain 3; LC3-II: microtubule-associated Protein 2 light chain 3 figure, *, indicates comparison with the control group, *P* < 0.05

Figure 3 shows that, compared with the normal control group, the control group not treated with T0901317 showed significantly decreased expression of P62 (*P* < 0.05), and increased LC3-I, LC3-II levels (all *P* < 0.05). These indicate that in the absence of T0901317 processing, the autophagy level was significantly lower in BTBR than the normal B6 mice. After T0901317 treatment, the brain of autistic mice had significantly higher P62 protein than the control group (*P* < 0.05), and significantly lower LC3-I and LC3-II levels than the control group (all *P* < 0.05).

### Comparisons on expressions of phosphorylated Ras/Raf ERK1/2 protein

**Fig. 4:**
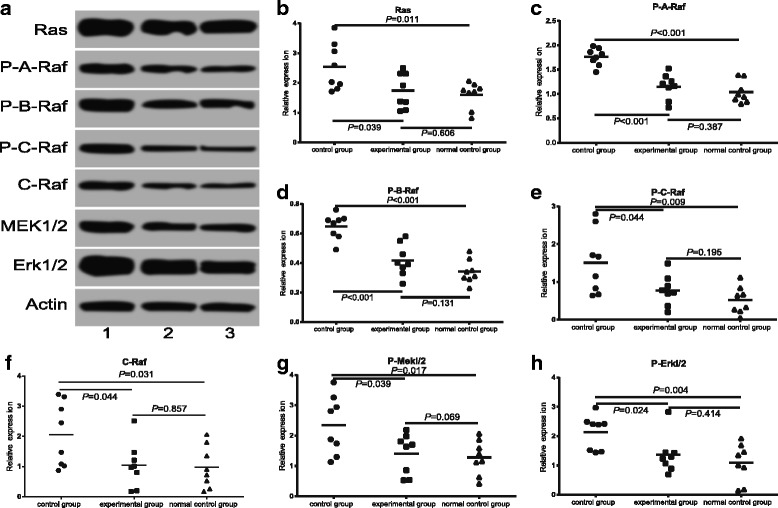
Expression of phosphorylated Ras/Raf/Mekl/2/Erkl/2 protein in cerebral cortex of three groups of mice. Note: (**a**) shows Western blot assay of cerebral cortex phosphorylated Ras/Raf/Mekl/2/Erkl/2 protein expressions in three groups of mice; (**b**-**h**) show cerebral cortex Ras, P-A-Raf, P-B-Raf, P-C-Raf, C-Raf, P-Mekl/2 and P-Erkl/2 expressions. In (**a**) 1 represents the control group; 2 the experimental group; 3, the normal control group. Ras: upstream activating protein of mitogen-activated protein kinases (MAPKs); C-Raf, P-A-Raf, P-B-Raf, P-C-Raf: Ser/Thr protein kinase of 40-75kD; P-Mekl/2: dual specificity protein kinase; P-Erkl/2: extracellular regulation protein kinase

Compared with the normal control group, control group without T0901317 treatment had significantly increased levels of related proteins (*P* < 0.05). Compared with the control group, BTBR mouse treated with agonist showed significantly weakened Ras, PA- Raf, PB-Raf, PC-Raf, C-Raf, P-Mekl/2, P-Erkl/2 bars, and reduced average expression (all *P* < 0.05), as shown in Fig. 4.

### Comparison of stereotyped behavior

Autistic mice showed sign of repeated combing, sustained grasping, licking, biting and other stereotypic movements. In order to observe the behaviors of mice with autism, we observed the activities of autistic mice within one hour via videos. The results are shown in Table 2. Compared with the normal control group, control group without T0901317 treatment showed significantly higher stereotyped behavior (rotation, rears, sniffing, grooming and exploratory activity) (all *P* < 0.05), suggesting that when not treated with T0901317, BTBR mice showed more severe repeated stereotyped behavior than normal B6 mice. After treatment of LXRβ agonist T0901317, repeated stereotyped behaviors was significantly improved in autistic mice; significant differences were found in comparisons of stereotyped behaviors between the experimental group and the control group (all *P* < 0.05), while no such difference was found between the experimental group and the normal control group (all *P >* 0.05).

**Table 2:** Ratings of stereotyped behavior among different groups of mice (% of Normal control)

Groups	*N*	Rotation	Rears	Sniffing	Grooming	Exploratory
Control group	8	182.13 ± 28.99	136.88 ± 12.92	154.50 ± 17.46	162.25 ± 34.69	148.38 ± 30.68
Experiment group	8	99.63 ± 11.71^*^	100.50 ± 7.76^*^	101.88 ± 7.79^*^	102.63 ± 8.03^*^	101.25 ± 8.15^*^
Normal control group	8	100.00 ± 0.00^*^	100.00 ± 0.00^*^	100.00 ± 0.00^*^	100.00 ± 0.00^*^	100.00 ± 0.00^*^

*indicates comparison with the control group, *P* < 0.05

### Comparisons of results of OP test

**Fig. 5:**
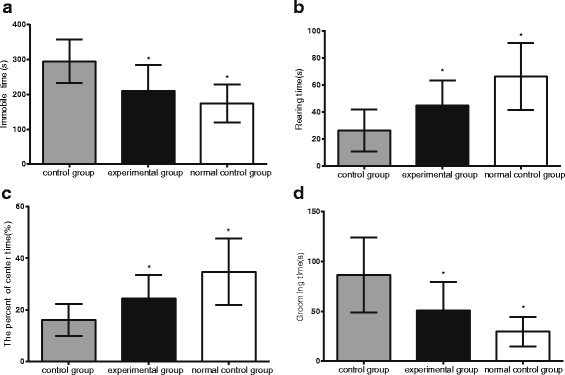
Comparison of inactivity, wall standing time, the percentage of time in central region, and self-combing time (not shown). Note: (**a**) shows comparison of inactivity among the three groups; (**b)** time spend standing facing the wall; (**c**) central area stay time; (**d**) self-combing time; *, represents comparison with the control group, *P* < 0.05

OP test was used to examine spontaneous activity and anxiety. Autism mice exhibited significant behavioral disorders and narrowed interests. Compared with the normal control group, mice in control group without T0901317 treatment showed significantly increased combing time, and decreased percentage of time in central stay and wall standing (all *P* < 0.05). Compared with control group without T0901317 treatment, mice treated with LXRβ agonist T0901317 showed significantly reduced inactivity time and self-combing time, and significantly increased percentage of time in central stay and wall standing (all *P* < 0.05). These results show that BTBR mice treated with T0901317 showed less autism symptoms: improvements in spontaneous activities, narrowed interests, stereotyped repetitive movements, lack of aversion and other behaviors (all *P* < 0.05) (Fig. 5).

### Comparisons on the results of water maze test

**Fig. 6:**
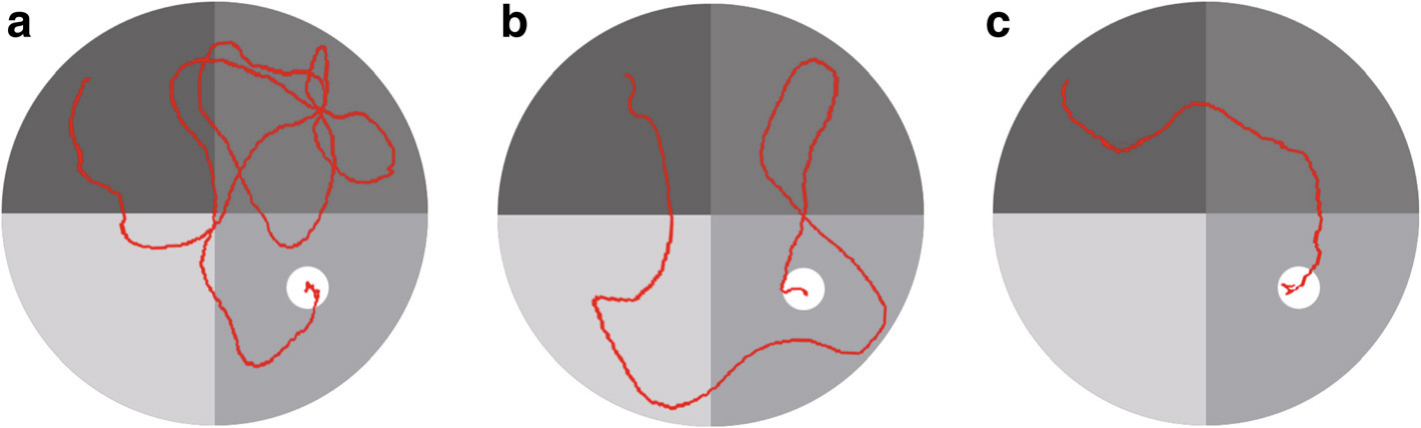
Water maze - space search experiment path in three groups of mice under navigation test. Note: (**a**) control group; (**b**) experimental group; (**c**) normal control group

In the navigation test, as shown in Fig. 6, normal B6 mice (C) used more straight path and relatively shorter time to reach the platform, while the control group (A) used more tortuous path and used longer time as compared with the normal control group (*P* < 0.05). BTBR mice treated with T0901317 (B) used a path similar with that of the control group (C), and their difference was not statistically significant (*P >* 0.05).

**Fig. 7:**
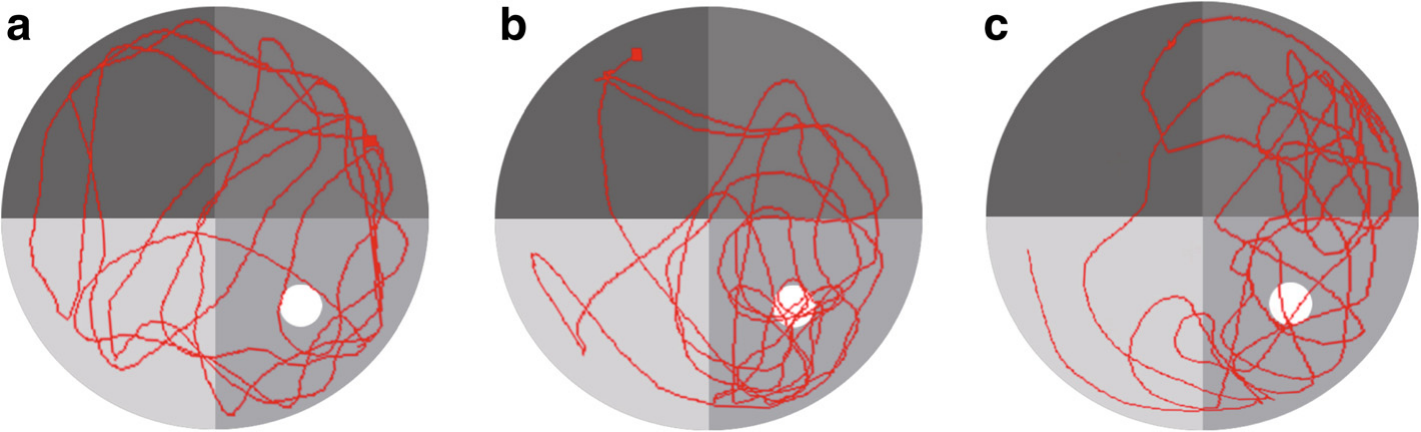
Water maze - space search experiment path in three groups of mice under space search. Note: (**a**) control group; (**b**) experimental group; (**c**) normal control group

The space search results (Table 3) showed that the T0901317 treated mice stayed longer in the platform quadrant and had higher number of platform crossing during the of 90 s space search experiment as compared with the control group (all *P* < 0.05), but similar to that of the normal control group (all *P >* 0.05). Figure 7 shows the space search paths for all groups, which suggests that autism mice treated with T0901317 (B) stayed mostly in the inner quadrant platform and crossed the platform more frequently, which was similar to that of the normal control group (C); the autistic mice (A) showed more scattered paths and fewer platform crossing.

**Table 3:** Comparison of water maze - space search experiments in different groups of mice

Groups	*N*	Quadrant swimming time (s)	Times of platform crossing
Control group	8	28.47 ± 8.15	1.25 ± 0.71
Experiment group	8	38.43 ± 7.56^*^	3.13 ± 1.64^*^
Normal control group	8	40.69 ± 7.12^*^	3.63 ± 1.41^*^

*indicates comparison with the control group, *P* < 0.05

## Discussion

Our presented study focused on the effect of LXRβ on Aβ peptide generation and autism behaviors. And we found that LXRβ could potentially reduce brain Aβ generation by inhibiting Aβ production and promoting Aβ degradation, thereby increasing the expression of autophagy-related proteins, reducing Ras/Raf/Erkl/2 signaling pathway proteins, and improving autism behaviors.

This study used T0901317 to activate LXRβ, and found NEP, IDE expression was significantly increased, BACE1 expression was downregulated, and Aβ expression was significantly decreased. A study conducted by Tan et al. has confirmed that LXR is one of the transcription factors that regulate the development of the cerebral cortex lamellar [21]. LXRβ plays an important role during development of the central nervous system, and in the development of a variety of neurodegenerative diseases, such as Alzheimer’s disease and Parkinson’s disease [22–24]. In addition, multiple studies have shown that mice with T0901317-activated LXR have lower cholesterol and Aβ levels in the brain, thereby alleviating dementia symptoms [25, 26]. Studies have shown, IDE may specifically bind to and degrade Aβ, and NEP is an in vivo catabolic rate-limiting enzyme of Aβ, the upregulation of these two proteins will reduce Aβ levels in human body [27, 28]. BACE1 protein is a membrane-bound aspartic protease and a rate-limiting enzyme to the APP cleavage that generates Aβ [29]. Past research has shown that activation of Erkl/2 protein pathway can significantly inhibit Aβ secretase enzyme expression [30, 31]. In addition, Aβ overproduction or excessive deposition can cause excessive activation of neighboring glial cells, even nerve cell death [15]. This mechanism might be a major cause of autism symptoms; therefore, when its level is lower the clinical symptoms of autism will be improved.

The study also observed that T0901317 can reduce the expression of Ras/Raf/Erkl/2 signaling pathway proteins. Ras/Raf/Erkl/2 signaling pathway is one of the mitogen-activated protein kinase family members, which is a hub in the cellular signal transduction network and an important path for the cell membrane receptor signals transmission, and involves in a variety of physiological processes including cell growth, development, division and death through regulation of cell transcription factors [32, 33]. Besides, Ras/Raf/Erkl/2 (extracellular signal-regulated kinase) signaling pathway is proved to play an important role in neuronal development as well as learning and memory [34]. Among these proteins, Ras is the upstream activating protein which is an important component of memory formation, Raf is a Ser/Thr protein kinases with the size of 40-75kD and has three subtypes: A-Raf, B-Raf, C-Raf (Raf −1), which can activate ERKs [35]. ERK is MAPK which includes five subtypes: Erkl/2, ERK5, P38, JNK, ERKl and ERK2. ERK is a key substance for signal conduction from surface receptor to the nucleus, mainly involves in the regulation of meiosis, mitosis and other cellular processes [36]. Ras/Raf/ERKl/2 pathway plays an important role in the generation of neural precursor cells, the formation and development of synaptic signaling neural crest, and is directly related to the formation of awareness, learning and memory, and can promote nerve cell death. Ras/Raf/ERK1/2 signaling activity may be increased in the autistic mice models [35]. Other studies have shown that the unregulated expression in multiple proteins in the Ras/Raf/ERKl/2 signal pathway may be one of the molecular mechanisms of autism [37].

## Conclusion

In summary, LXRβ can inhibit Aβ production and enhance Aβ degradation, thereby reducing brain Aβ generation, increasing the expression of autophagy-related proteins, reducing the expression of Ras/Raf/Erkl/2 signaling pathway proteins, and eventually improving autism symptoms. Our findings open up new ideas for autism treatment. However, there are also some limitations in our study: firstly, there are various factors affecting the construction of model, including various factors affecting the incidence of autism, the specificity of the clinical manifestations and the particularity of biological gene; so it is hard to replicate a model that is exactly the same as human autism. And, in view of the limitations of animal model, it is necessary to be cautious when it is extended to human patients with autism; secondly, LXR agonist T0901317 can activated both LXRα and LXRβ, while, the main purpose of our study was to investigate the relationship between LXRβ and ASD, so the roles of LXRα and LXRβ activated by LXR agonist on ASD need to be further explored; thirdly, we cannot observe the specific changes of the indicators before and after treatment in the same mice because we selected the brain tissue of mice for our experiment.

## References

[1] Geschwind DH (2011). Genetics of autism spectrum disorders. Trends Cogn Sci.

[2] Tchaconas A, Adesman A (2013). Autism spectrum disorders: a pediatric overview and update. Curr Opin Pediatr.

[3] Li N, Chen G, Song X, Du W, Zheng X (2011). Prevalence of autism-caused disability among Chinese children: a national population-based survey. Epilepsy Behav.

[4] Ha S, Sohn IJ, Kim N, Sim HJ, Cheon KA (2015). Characteristics of Brains in Autism Spectrum Disorder: Structure, Function and Connectivity across the Lifespan. Exp Neurobiol.

[5] Heyvaert M, Saenen L, Campbell JM, Maes B, Onghena P (2014). Efficacy of behavioral interventions for reducing problem behavior in persons with autism: an updated quantitative synthesis of single-subject research. Res Dev Disabil.

[6] Durkin MS, Elsabbagh M, Barbaro J, Gladstone M, Happe F, Hoekstra RA (2015). Autism screening and diagnosis in low resource settings: Challenges and opportunities to enhance research and services worldwide. Autism Res.

[7] Matelski L, Van de Water J. Risk factors in autism: Thinking outside the brain. J Autoimmun. 2015;67:1–7 10.1016/j.jaut.2015.11.003 PMC5467975 26725748

[8] Theofilopoulos S, Arenas E (2015). Liver X receptors and cholesterol metabolism: role in ventral midbrain development and neurodegeneration. F1000Prime Rep.

[9] Sodhi RK, Singh N (2013). Liver X receptors: emerging therapeutic targets for Alzheimer’s disease. Pharmacol Res.

[10] Baranowski M (2008). Biological role of liver X receptors. J Physiol Pharmacol.

[11] Fan X, Kim HJ, Bouton D, Warner M, Gustafsson JA (2008). Expression of liver X receptor beta is essential for formation of superficial cortical layers and migration of later-born neurons. Proc Natl Acad Sci U S A.

[12] Warner M, Gustafsson JA (2015). Estrogen receptor beta and Liver X receptor beta: biology and therapeutic potential in CNS diseases. Mol Psychiatry.

[13] Barry DS, Pakan JM, McDermott KW (2014). Radial glial cells: key organisers in CNS development. Int J Biochem Cell Biol.

[14] Koldamova RP, Lefterov IM, Staufenbiel M, Wolfe D, Huang S, Glorioso JC (2005). The liver X receptor ligand T0901317 decreases amyloid beta production in vitro and in a mouse model of Alzheimer’s disease. J Biol Chem.

[15] Sun X, Chen WD, Wang YD (2015). beta-Amyloid: the key peptide in the pathogenesis of Alzheimer’s disease. Front Pharmacol.

[16] Korsak M, Kozyreva T (2015). Beta Amyloid Hallmarks: From Intrinsically Disordered Proteins to Alzheimer’s Disease. Adv Exp Med Biol.

[17] Haass C, Selkoe DJ (2007). Soluble protein oligomers in neurodegeneration: lessons from the Alzheimer’s amyloid beta-peptide. Nat Rev Mol Cell Biol.

[18] Yu J, Ryan DG, Getsios S, Oliveira-Fernandes M, Fatima A, Lavker RM (2008). MicroRNA-184 antagonizes microRNA-205 to maintain SHIP2 levels in epithelia. Proc Natl Acad Sci U S A.

[19] Yu J, Peng H, Ruan Q, Fatima A, Getsios S, Lavker RM (2010). MicroRNA-205 promotes keratinocyte migration via the lipid phosphatase SHIP2. FASEB J.

[20] Kelley AE (2001). Measurement of rodent stereotyped behavior. Curr Protoc Neurosci.

[21] Tan XJ, Fan XT, Kim HJ, Butler R, Webb P, Warner M (2010). Liver X receptor beta and thyroid hormone receptor alpha in brain cortical layering. Proc Natl Acad Sci U S A.

[22] Infante J, Rodriguez-Rodriguez E, Mateo I, Llorca J, Vazquez-Higuera JL, Berciano J (2010). Gene-gene interaction between heme oxygenase-1 and liver X receptor-beta and Alzheimer’s disease risk. Neurobiol Aging.

[23] Sacchetti P, Sousa KM, Hall AC, Liste I, Steffensen KR, Theofilopoulos S (2009). Liver X receptors and oxysterols promote ventral midbrain neurogenesis in vivo and in human embryonic stem cells. Cell Stem Cell.

[24] Koldamova R, Lefterov I (2007). Role of LXR and ABCA1 in the pathogenesis of Alzheimer’s disease - implications for a new therapeutic approach. Curr Alzheimer Res.

[25] Fitz NF, Cronican A, Pham T, Fogg A, Fauq AH, Chapman R (2010). Liver X receptor agonist treatment ameliorates amyloid pathology and memory deficits caused by high-fat diet in APP23 mice. J Neurosci.

[26] Lefterov I, Bookout A, Wang Z, Staufenbiel M, Mangelsdorf D, Koldamova R (2007). Expression profiling in APP23 mouse brain: inhibition of Abeta amyloidosis and inflammation in response to LXR agonist treatment. Mol Neurodegener.

[27] Zhao Z, Xiang Z, Haroutunian V, Buxbaum JD, Stetka B, Pasinetti GM (2007). Insulin degrading enzyme activity selectively decreases in the hippocampal formation of cases at high risk to develop Alzheimer’s disease. Neurobiol Aging.

[28] Wang S, Wang R, Chen L, Bennett DA, Dickson DW, Wang DS (2010). Expression and functional profiling of neprilysin, insulin-degrading enzyme, and endothelin-converting enzyme in prospectively studied elderly and Alzheimer’s brain. J Neurochem.

[29] Devi L, Ohno M (2015). Effects of BACE1 haploinsufficiency on APP processing and Abeta concentrations in male and female 5XFAD Alzheimer mice at different disease stages. Neuroscience.

[30] Sawamura N, Ko M, Yu W, Zou K, Hanada K, Suzuki T (2004). Modulation of amyloid precursor protein cleavage by cellular sphingolipids. J Biol Chem.

[31] Tamagno E, Guglielmotto M, Giliberto L, Vitali A, Borghi R, Autelli R (2009). JNK and ERK1/2 pathways have a dual opposite effect on the expression of BACE1. Neurobiol Aging.

[32] Heidorn SJ, Milagre C, Whittaker S, Nourry A, Niculescu-Duvas I, Dhomen N (2010). Kinase-dead BRAF and oncogenic RAS cooperate to drive tumor progression through CRAF. Cell.

[33] Davis S, Laroche S (2006). Mitogen-activated protein kinase/extracellular regulated kinase signalling and memory stabilization: a review. Genes Brain Behav.

[34] Yang K, Sheikh AM, Malik M, Wen G, Zou H, Brown WT (2011). Upregulation of Ras/Raf/ERK1/2 signaling and ERK5 in the brain of autistic subjects. Genes Brain Behav.

[35] Zou H, Yu Y, Sheikh AM, Malik M, Yang K, Wen G (2011). Association of upregulated Ras/Raf/ERK1/2 signaling with autism. Genes Brain Behav.

[36] Murphy LO, Blenis J (2006). MAPK signal specificity: the right place at the right time. Trends Biochem Sci.

[37] Yin A, Qiu Y, Jia B, Song T, Yu Y, Alberts I (2014). The developmental pattern of the RAS/RAF/Erk1/2 pathway in the BTBR autism mouse model. Int J Dev Neurosci.

